# Overcoming PCOS-Related Infertility by Using In Vitro Maturation Approach: A Case Report

**DOI:** 10.7759/cureus.62965

**Published:** 2024-06-23

**Authors:** Ayshwarya Jain, Pranita A Bawaskar, Nancy Nair, Avanti Kalbande, Charu Pareek

**Affiliations:** 1 Clinical Embryology, Datta Meghe Institute of Higher Education and Research (Deemed to be University), Wardha, IND; 2 Obstetrics and Gynaecology, Datta Meghe Institute of Higher Education and Research (Deemed to be University), Wardha, IND

**Keywords:** intracytoplasmic sperm injection, in vitro maturation, in vitro fertilization, polycystic ovarian syndrome, female infertility

## Abstract

Polycystic ovary syndrome (PCOS) is an endocrinological disorder affecting women of reproductive age, characterized by hormonal imbalance leading to metabolic and reproductive dysregulations. This case report revolves around a 30-year-old husband and his 27-year-old partner. The male partner had normozoospermia, and the female spouse had PCOS, according to the couple's diagnostic evaluations. The female patient received ovarian stimulation specifically to assist with PCOS, and the retrieved oocytes were then matured in vitro. After intracytoplasmic sperm injection (ICSI), fertilization and embryonic development were successful. Treatment of PCOS-related infertility presents many challenges, and in vitro maturation (IVM) and its potential as an effective assisted fertility method are discussed. To optimize treatment outcomes, the conclusion shows the importance of IVM and other assisted reproductive techniques for infertility. It also focuses on the necessary continuous research and clinical experience. Clinical pregnancy was confirmed by measuring serum beta-human chorionic gonadotropin (β-hCG) levels followed by ultrasound sonography (USG), which showed a normal growth rate of the fetus.

## Introduction

Polycystic ovary syndrome (PCOS) is a hormonal disorder that can cause infertility in women of childbearing age, in which 80% of cases are related to PCOS [1]. The irregular menstrual cycle, hyperandrogenism, and multiple cysts in the ovary, along with hirsutism, acne, alopecia, and infertility, are clinical manifestations of PCOS [2]. Female infertility can result from a variety of factors, including PCOS, hormonal disorders, premature ovarian failure, genital infections, endometriosis, fallopian tube obstruction, and congenital uterine anomalies [3]. Hyperandrogenism, when combined with hypothalamic-pituitary dysfunction, causes additional ovarian dysfunction, which can lead to anovulation and infertility. Women with PCOS may be at greater risk of miscarriage and develop complications during pregnancy, such as gestational diabetes [4].

In vitro maturation (IVM) implies the maturation of retrieved immature oocytes in a specific culture environment. In vitro-developed oocytes were used in the first human vitro fertilization effort as early as in vitro fertilization (IVF). IVM has much broader indications, such as poor ovarian reserve and multiple IVF failures [5]. IVM drew focus from fertility specialists due to its safety, recurrence affordability, low risk of ovarian hyperstimulation syndrome (OHSS), and suitable pregnancy rates. This medium is less expensive, safer, and more convenient for patients because it requires fewer hormone injections, shorter stimulation cycles, prevents OHSS, and requires less frequent monitoring [6]. The earliest reports of human conception in culture verified that when removed from follicles and put in the proper culture medium, human follicular oocytes may develop in vitro [7]. The maturation rate of human oocytes surrounded by cumulus cells is more significant. The production of a positive signal required to complete oocyte maturation in vivo coincides with rising follicle-stimulating hormone (FSH) levels within the follicle [8].

Recombinant follicle-stimulating hormone (rFSH) follows a long down-regulation protocol in IVF intracytoplasmic sperm injection (ICSI) treatment cycles [9]. After oocytes are collected, meiosis begins again by administering a human chorionic gonadotrophin (hCG) trigger, known as hCG priming. Oocytes that may be at different stages of maturation are germinal vesicles (GV), metaphase I (MI), or metaphase II (MII) collected [10]. IVM in patients with PCOS can eliminate the risk of OHSS [11].

## Case presentation

Patient information

A 27-year-old female and her 30-year-old husband have been married for five years. After trying to conceive naturally for three and a half years, the couple decided to go for assisted reproductive technology (ART) and was referred to our clinic. The woman worked as an employee, while her husband was a businessman. The patient's chief complaint was an irregular menstrual cycle. After detailed counseling, the patient insisted on going for IVF.

Medical history

For the past three and a half years, the couple has been engaging in regular and unprotected sexual intercourse, but they have failed to achieve a successful pregnancy. The male partner has been diagnosed with normozoospermia, indicating normal sperm parameters in semen analysis and no documented issues with conception, suggesting that the fertility issue likely lies with the female partner. The female partner has been diagnosed with PCOS by gynecologists after two years of marriage. There is no significant history of inherited conditions among immediate family members with hypertension, diabetes, thyroid, or prior surgeries in partners. The male patient maintains a healthy lifestyle, avoiding smoking, tobacco, and alcohol consumption.

Clinical findings

The physical health of the male patient seems normal. The male partner semen investigation showed that the progressive motility was 42%, with a count of 120 million/mL of ejaculation, which is within the normal range as stated in the World Health Organization's WHO 2021 guidelines [12], as shown in Table 1.

**Table 1:** Semen analysis report. pH: potential of hydrogen, ml: milliliter

Semen parameters	Findings	Reference range
Ejaculatory abstinence	5 days	2-7 days
Volume	2.2ml	1.4ml
Appearance	Opalescent gray	Opalescent gray
pH	7.8	7.2-8.0
Sperm count	120 million/ml	16 million/ml
Total sperm motility	60%	42%
Progressive motility	42%	30%
Morphology	6%	>4%

Diagnostic assessment

The female patient appeared to be obese, with a body mass index (BMI) of more than 29 kg/m². The heart rate, blood pressure, and respiration rate were normal. The FSH value was 11.5 mIU/mL. The luteinizing hormone (LH) was 26.5 mIU/mL. Estradiol, an important form of estrogen, is at 74 pg/mL. Testosterone levels are low at 2.6 ng/mL. The anti-Mullerian hormone (AMH) test level was 1.5 ng/mL. The progesterone level is at 16 ng/mL. The antral follicle count (AFC) of both ovaries is 9. The thyroid-stimulating hormone (TSH) level was 2.37 mIU/mL. The prolactin level was 24.88 ng/mL, as mentioned in Table 2.

**Table 2:** Complete hormonal profile of the female patient. mIU/mL: milli international units per milliliter, pg/mL: picograms per milliliter, nmol/L: nanomole per liter, ng/ml: nanograms per milliliter

Parameters	Findings	Reference range
Follicle-stimulating hormone	11.5 mIU/mL	3.5-12.5 mIU/mL
Luteinizing hormone	26.5 mIU/mL	5-15 mIU/mL
Estradiol	74 pg/mL	25-450 pg/mL
Testosterone	2.6 ng/mL	0.15-0.7 ng/mL
Anti-Mullerian hormone	1.5 ng/mL	1.0-4.0 ng/mL
Progesterone	16 ng/mL	1-8 ng/mL
Antral follicular count (left + right)	9	10-11
Prolactin	24.88 ng/mL	80-400 ng/mL
Thyroid-stimulating hormone	2.37 mIU/mL	0.4-4.0 mIU/mL

**Figure 1:**
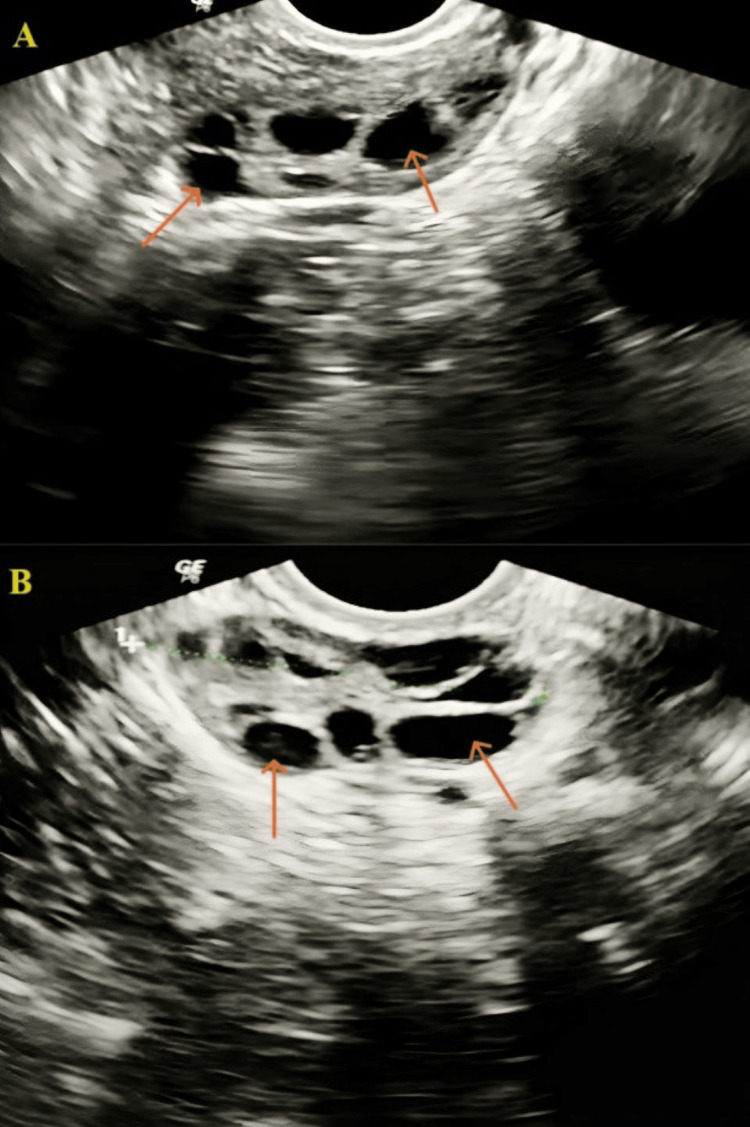
Transvaginal sonography image of the patient A: right ovary, B: left ovary. The orange arrow shows the ovarian cyst.

To confirm the diagnosis of PCOS, a transvaginal ultrasound (TVS) was performed, and AFC was found to be nine, within the reference range, as shown in Figure 1. 

Therapeutic intervention

Stimulation Protocol

The female partner received a gonadotropin-releasing hormone (GnRH) antagonist protocol for ovarian stimulation along with PCOS, focusing on maximizing ovulation and follicular growth. Starting on the second day of menstruation, the treatment included 100 mg of clomiphene citrate and 150 IU of rFSH. To prevent early ovulation, cetrotide 0.25 mg daily was added on the seventh day of the regimen and continued until day 11, and 0.25 mg of decapeptyl was administered as a trigger on day 14 to manage the cycle further and reduce endogenous gonadotropin secretion. After 36 hours, the oocytes were retrieved.

ICSI Procedure

**Figure 2:**
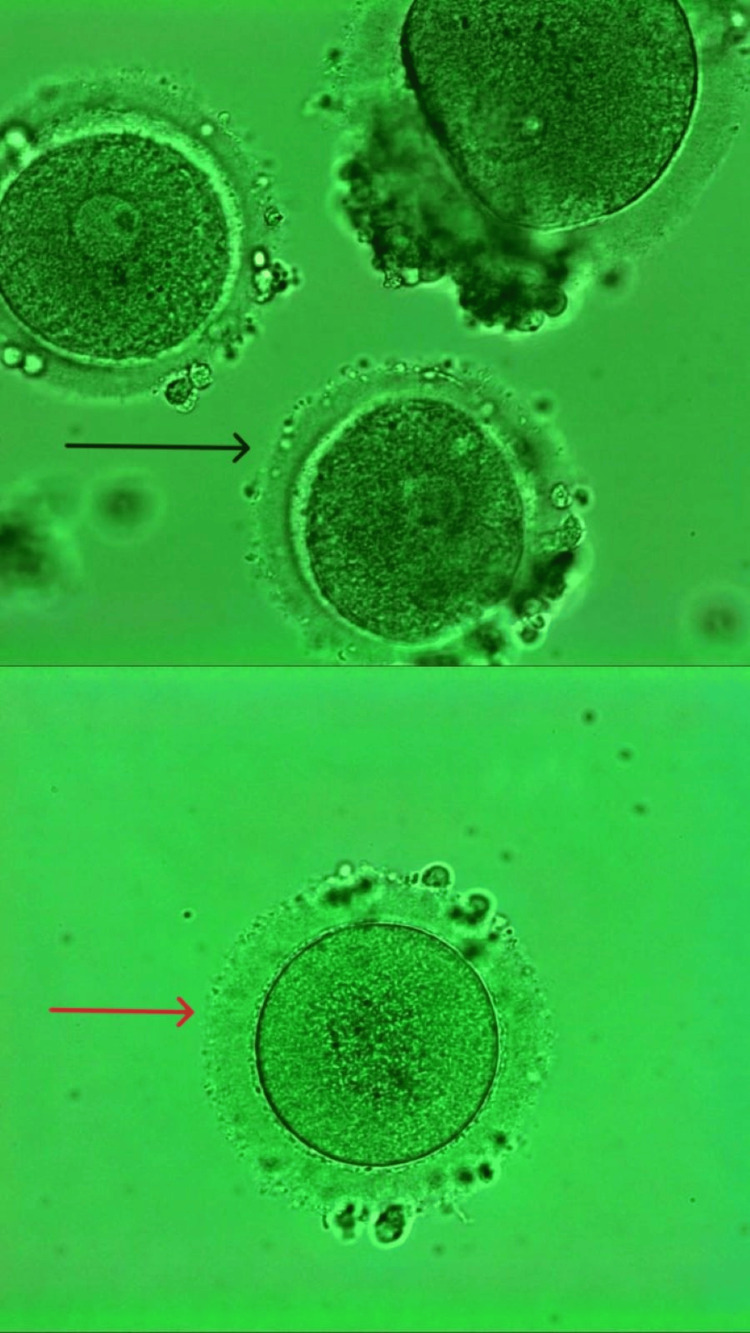
Retrieval of immature oocytes after ovum pickup. The black arrow shows the oocyte at the germinal vesicle stage. The red arrow shows the oocyte at the metaphase I stage.

Following oocyte pick-up (OPU), nine oocytes were retrieved, three were at the GV stage, and six were at the MI stage. No oocytes were retrieved at the MII stage. The IVM medium involves a controlled environment for the growth and development of embryos. Retrieved immature oocytes were placed in a specialized culture medium designed to support oocyte maturation. Oocytes were incubated at 37°C in a controlled environment (usually 5% CO_2_) for 24-48 hours. The medium contained hormones, such as FSH, LH, and estradiol, along with electrolyte solutions, bicarbonates, and energy substrates, such as glucose and pyruvate essential and nonessential amino acid, and protein supplements, such as human serum albumin (HSA) or plasma protein fraction. During the incubation period, the oocytes were monitored for maturation. Out of the nine retrieved oocytes, four were matured into the MII stage. Following this, four oocytes were fertilized by the ICSI process. Two embryos were in the eight-cell stage by day 3 of embryonic development, while one embryo was in the six-cell stage. One embryo reached the 12-cell stage by day 4, while another had developed the 14-cell stage. Out of a total of nine retrieved embryos, two of day 4 embryos were cryopreserved. After two months, cryopreserved embryos were thawed and transferred in a single frozen embryo transfer (FET) cycle. After the embryos were transferred, the patient was advised to rest, avoid strenuous activity, and come for a follow-up after 14 days. Figure 2 shows the retrieved GV and MI after ovum pickup.

Follow-up

The doctor prescribed medications, such as progesterone, iron, calcium supplements, and multivitamins. They were advised to come for a follow-up visit after two weeks. Biochemical pregnancy was confirmed by monitoring the levels of serum beta-human chorionic gonadotropin (β-hCG), which were 798 mIU/mL. An ultrasound was done to examine the normal growth rate of the fetus.

## Discussion

The case study highlights the difficulties in treating PCOS-related infertility and emphasizes the need to make lifestyle changes as the initial line of treatment. Furthermore, the newly developed method of oocyte maturation shows potential as an easy and secure means of assisted reproduction, especially for individuals who have poor oocyte maturation or who are at risk. In AFC-elevated patients, abnormal ovulation or anovulation is present. In PCOS patients, the AFC level is elevated, and PCOS patients are considered for IVM treatment.

Hatırnaz et al. stated that IVM was initially prescribed for PCOS and OHSS patients who had undergone prior IVF procedures. Still, in recent years, its indications have increased to nearly all forms of infertility. Managing PCOS-related problems by changing lifestyle habits and using medication followed by ART is necessary to resolve infertility [13]. According to Tannus et al., women undergoing IVM treatment experienced a clinical pregnancy rate of 44.7% and a live birth rate of 34.6%, with most transfers involving a single embryo [14]. Jaroudi et al. reported on 18 patients initially undergoing IVF who were later identified as being at high risk for developing OHSS. Their cycles were canceled, and they underwent immature oocyte retrieval followed by IVM. On average, each patient had 8.1 immature oocytes retrieved, and a total of 44 embryos were transferred across 17 cycles. The study indicated that oocytes matured in vitro from incomplete IVF cycles could be fertilized using ICSI and that these embryos could potentially lead to pregnancies [15].

Yang et al. concluded that using a biphasic IVM culturing technique, culture media, and supplements significantly impacts the IVM results. Certain factors, such as various follicular priming techniques, various protein sources and hormones in the medium, and different culture media, have an unknown effect on IVM [16].

Jing et al. concluded in their study that, in addition to thousands of healthy births, significant improvements in pregnancy and implantation rates have been made possible by IVM technology. IVM increases the usage of this technique and enhances patient benefits, especially when combined with other treatments. The best ways to increase the effectiveness of IVM and make it applicable to a larger population are to gain a deeper comprehension of the whole molecular process of oocyte maturation and to adopt the most influential culture techniques for multiple stages of oocytes formed through different methods [17].

## Conclusions

The efficacy of ART has been observed, especially IVM of oocytes in treating PCOS-related infertility. Despite the challenges brought on by PCOS-related ovulatory dysfunction, IVM combined with PCOS-specific ovarian stimulation techniques resulted in successful conception and embryonic development. For PCOS patients, particularly those who are at risk of poor oocyte maturation or OHSS, further research and clinical experience will continue to optimize and improve effectiveness.

## References

[1] Yang L, Liang F, Yuan Y, Luo X, Wang Q, Yao L, Zhang X (2023). Efficacy of progestin-primed ovarian stimulation in women with polycystic ovary syndrome undergoing in vitro fertilization: a systematic review and meta-analysis. Front Endocrinol (Lausanne).

[2] Shrivastava R, Pathak T, Shrivastava P (2023). Assessment of cardiac autonomic function in women with polycystic ovary syndrome through Ewing’s battery, heart rate variability analysis, and composite autonomic symptom score-31 scale. Cureus.

[3] Benksim A, Elkhoudri N, Addi RA, Baali A, Cherkaoui M (2018). Difference between primary and secondary infertility in Morocco: frequencies and associated factors. Int J Fertil Steril.

[4] Cunha A, Póvoa AM (2021). Infertility management in women with polycystic ovary syndrome: a review. Porto Biomed J.

[5] Das M, Son WY (2023). In vitro maturation (IVM) of human immature oocytes: is it still relevant?. Reprod Biol Endocrinol.

[6] Yang H, Kolben T, Meister S (2021). Factors influencing the in vitro maturation (IVM) of human oocyte. Biomedicines.

[7] Telfer EE, Andersen CY (2021). In vitro growth and maturation of primordial follicles and immature oocytes. Fertil Steril.

[8] Zhang A, Xu B, Sun Y (2012). The effect of human cumulus cells on the maturation and developmental potential of immature oocytes in ICSI cycles. J Assist Reprod Genet.

[9] Figen Turkcapar A, Seckin B, Onalan G, Ozdener T, Batioglu S (2013). Human menopausal gonadotropin versus recombinant FSH in polycystic ovary syndrome patients undergoing in vitro fertilization. Int J Fertil Steril.

[10] Julania S, Walls ML, Hart R (2018). The place of in vitro maturation in PCO/PCOS. Int J Endocrinol.

[11] Xu Y, Qiao J (2021). Comparison of in vitro maturation and in vitro fertilization for polycystic ovary syndrome patients: a systematic review and meta-analysis. Ann Transl Med.

[12] (2024). WHO laboratory manual for the examination and processing of human semen. https://www.who.int/publications-detail-redirect/9789240030787.

[13] Hatırnaz Ş, Ata B, Hatırnaz ES, Dahan MH, Tannus S, Tan J, Tan SL (2018). Oocyte in vitro maturation: a sytematic review. Turk J Obstet Gynecol.

[14] Tannus S, Hatirnaz S, Tan J (2018). Predictive factors for live birth after in vitro maturation of oocytes in women with polycystic ovary syndrome. Arch Gynecol Obstet.

[15] Jaroudi KA, Hollanders JM, Elnour AM, Roca GL, Atared AM, Coskun S (1999). Embryo development and pregnancies from in-vitro matured and fertilized human oocytes. Hum Reprod.

[16] Yang Q, Zhu L, Jin L (2020). Human follicle in vitro culture including activation, growth, and maturation: a review of research progress. Front Endocrinol (Lausanne).

[17] Jing YX, Wang YQ, Li HX, Yue F, Xue SL, Zhang XH (2021). Research progress of in vitro oocyte maturation. Reprod Dev Med.

